# Role of N-Linked Glycosylation in PKR2 Trafficking and Signaling

**DOI:** 10.3389/fnins.2021.730417

**Published:** 2021-08-13

**Authors:** Jissele A. Verdinez, Julien A. Sebag

**Affiliations:** ^1^Department of Molecular Physiology and Biophysics, Carver College of Medicine, University of Iowa, Iowa City, IA, United States; ^2^Fraternal Order of Eagles Diabetes Research Center, University of Iowa, Iowa City, IA, United States; ^3^Iowa Neuroscience Institute, University of Iowa, Iowa City, IA, United States

**Keywords:** prokineticin receptor, GPCR, MRAP2, trafficking, glycosylation

## Abstract

Prokineticin receptors are GPCRs involved in several physiological processes including the regulation of energy homeostasis, nociception, and reproductive function. PKRs are inhibited by the endogenous accessory protein MRAP2 which prevents them from trafficking to the plasma membrane. Very little is known about the importance of post-translational modification of PKRs and their role in receptor trafficking and signaling. Here we identify 2 N-linked glycosylation sites within the N-terminal region of PKR2 and demonstrate that glycosylation of PKR2 at position 27 is important for its plasma membrane localization and signaling. Additionally, we show that glycosylation at position 7 results in a decrease in PKR2 signaling through Gα_s_ without impairing Gα_q/__11_ signaling.

## Introduction

Prokineticin receptors (PKRs) are GPCRs expressed both centrally and peripherally (Cheng et al., 2006; Maldonado-Perez et al., 2007; Mohsen et al., 2017). The two members of the family (PKR1 and PKR2) couple both to Gα_q/__11_ and Gα_s_ (Chaly et al., 2016; Rouault et al., 2017a). In the periphery, PKRs agonists are known to modulate nociception through regulation of TRPV1 (Hu et al., 2006; Negri et al., 2006; Negri and Lattanzi, 2011; Qiu et al., 2012; Maftei et al., 2020). In the brain, the PKR agonist prokineticin-2 (PK2) acts to decrease food intake in a melanocortin independent manner (Gardiner et al., 2010; Beale et al., 2013; Chaly et al., 2016), however, the mechanism involved is still unclear. Both PKR1 and PKR2 are inhibited by the Melanocortin Receptor Accessory Protein 2 (MRAP2) (Chaly et al., 2016; Rouault et al., 2017a), a single transmembrane protein forming anti-parallel homodimers which has been shown to regulate several GPCRs involved in the control of energy homeostasis (Asai et al., 2013; Sebag et al., 2013; Chaly et al., 2016; Rouault et al., 2017a,b, 2020; Srisai et al., 2017; Bruschetta et al., 2018; Yin et al., 2020; da Fonseca et al., 2021). MRAP2 traps PKRs in intracellular compartments and inhibits their signaling (Chaly et al., 2016; Rouault et al., 2017a). The potent suppression of feeding by PK2 represents a promising novel approach to regulate appetite and possibly a new target for the treatment of obesity, for this reason, it is important to understand how the trafficking and signaling of PKRs are regulated. GPCR trafficking is regulated by numerous factors including homo- and hetero-dimerization, accessory proteins and post-translational modifications. One such post-translational modification is N-linked glycosylation which has been shown to be important for proper targeting of several GPCRs to the plasma membrane (Duvernay et al., 2005; Patwardhan et al., 2021). While PKR2 possesses two putative N-linked glycosylation sites at positions N7 and N27, their role and importance has not yet been established. In this study, we assessed the role of PKR2 glycosylation at both putative sites for receptor localization and signaling and the effect of MRAP2 on PKR2 glycosylation.

## Materials and Methods

### Cell Culture and Transfection

CHO-K1 were cultured in Dulbecco’s modified Eagle’s medium (DMEM)/F-12 (Thermo Fisher Scientific) supplemented with 5% (v/v) of a 1:1 mix of calf bovine serum and fetal bovine serum and 1% penicillin-streptomycin. Cells were incubated at 37°C in a humidified atmosphere consisting of 5% CO_2_. Cells were transfected with LipoD293 DNA transfection reagent (SignaGen Laboratories #SL100668) following manufacturer’s instructions or with PEI transfection reagent (Polysciences Inc.).

### Plasmids

PKR2 plasmid was obtained from the Missouri S&T cDNA Resource Center. The N-terminal 2HA-tag was added by PCR amplification as previously described (Chaly et al., 2016). The PKR2-GFP plasmid was generated by amplifying PKR2 and GFP sequences separately and assembling them into a pcDNA3.1 + vector using the HiFi DNA assembly kit (NEB). N7Q and N27Q mutations in 2HA-PKR2 and PKR2-GFP were generated by site directed mutagenesis using the Q5-site directed mutagenesis kit (NEB). Cloning and mutations were verified by sequencing. Generation of hMRAP2-3XFlag was previously described (Sebag and Hinkle, 2010). pGloSensor^TM^-22F plasmid was obtained from Promega.

### Co-immunoprecipitation

CHO-K1 cells were seeded in 6 well plates were transfected with indicated plasmids using LipoD293 transfection reagent. Cells were lysed in 0.1% n-dodecyl-β-maltoside and protease inhibitor cocktail (Sigma catalog no. P8340) in PBS at 4°C. Lysates were centrifuged 10 min at 10000 rpm 4°C and supernatants were incubated with either 20 μl GFP-nanobody agarose resin or a 1:5000 dilution of M2 Anti-Flag mouse monoclonal antibody overnight at 4°C. Immune complexes bound to anti-Flag antibody were precipitated with Dynabeads Protein G (Life Technologies #10003D) at 4°C for 1 h. Beads were washed 3 times with ice cold lysis buffer. Samples were resuspended in LDS loading buffer with 5% β-mercaptoethanol and boiled for 5 min.

### Immunoprecipitation

CHO-K1 cells were seeded in 60 mm dishes and transfected with the indicated plasmids. Cells were lysed in RIPA buffer supplemented with protease inhibitor cocktail. Lysates were centrifuged and supernatants were incubated with GFP-nanobody agarose resin for 1 h at 4°C. Nanobody complexes were washed three times with lysis buffer and resuspended in LDS loading buffer containing 5% β-mercaptoethanol and boiled for 5 min.

### Western Blot

Proteins were resolved by SDS/PAGE and transferred to PVDF membrane. Membranes were blocked for 1 h in 5% non-fat dry milk in PBST, incubated with either mouse anti-flag at 1:5000 or rabbit anti-GFP (Cell Signaling Technologies #29565) at 1:2000 in blocking buffer o/n 4°C, washed three times 5 min in PBST, incubated with either anti-mouse or anti-rabbit-HRP secondary antibody in blocking buffer for 1 h RT, washed three times and imaged using ECL substrate and iBright imager. Densitometry analysis was conducted using photoshop.

### Glycosidase Treatments

CHO-K1 cells were seeded in 60 mm dishes and transfected with PKR2-GFP with or without MRAP2 using LipoD293 transfection reagent. Cells were lysed in RIPA bufer supplemented with protease inhibitor cocktail. Lysates were centrifuged 10 min at 10000 rpm 4°C and supernatants were incubated with GFP nanobody coated beads for 1 h at 4°C. Beads were washed three times in lysis buffer. For PNGase F treatment, samples were resuspended in 10 μl glycoprotein denaturing buffer (NEB PNGase F P0708S) and heated at 60°C for 10 min. PNGase F reaction mixture was prepared and added to reaction using 2 μl of GlycoBuffer 2, 2 μl of 10% NP-40, 1 μl PNGase F, and 5 μl water. Samples were incubated at 37°C for 1 h. Following incubation, 30 μl of 2 × LDS loading buffer with 5% β-mercaptoethanol was added to each sample. For Endo H treatment, samples were resuspended in 10 μl glycoprotein denaturing buffer (NEB Endo H P0702S) and heated at 60°C for 10 min. Endo H reaction mixture containing 2 μl of Glycobuffer 3, 1 μl Endo H and 7 μl milliq water was added to samples and incubated at 37°C for 1 h. Following incubation, 30 μl 2 × LDS loading buffer with 5% β-mercaptoethanol was added to each sample.

### Fixed Cell ELISA

CHO-K1 cells were plated in 24 well plates, and triplicate wells were transfected with empty vector, 2HA-PKR2, 2HA PKR2 N7Q, 2HA-PKR2 N27Q, and 2HA-PKR2 N7/27Q. 24 h following transfection, cells were rinsed with PBS and fixed for 10 min in 4% paraformaldehyde (PFA) in PBS. PFA was washed three times with PBS, and cells were blocked with 5% non-fat dry milk in PBS or RIPA for 30 min at room temperature on a shaker. Cells were incubated with anti-HA antibody at 1:5000 in blocking buffer for 2 h at room temperature on a shaker. Cells were washed three times with PBS for 5 min at room temperature on a shaker and then incubated with anti-mouse HRP at 1:5000 in blocking buffer for 1 h at room temperature on a shaker. Cells were washed three times for 5 min with PBS. 200 μl 3,3′,5,5′-tetramethylbenzidine (Sigma-Aldrich) was then added until blue color was visible and the reaction was stopped with 200 μl 10% sulfuric acid. 300 μl of each sample was transferred to a 96 well plate, and absorbance was measured at 450 nm using a Spectramax i3 plate reader (Molecular Devices, Sunnyvale, CA). Each condition was run in triplicate, and experiments were repeated independently at least three times.

### Inositol Phosphate Assay

CHO-K1 cells were seeded in six-well plates and transfected with the indicated plasmids. Cells were then lifted using TrypLE Express (Thermo Fisher Scientific) and resuspended in 400 μl DMEM/F12. Cell suspension (7 μl/well) was transferred to a white opaque 384-well plate before adding agonist in stimulation buffer (included in kit). Cells were incubated for 1 h at 37°C, lysed and accumulated IP_1_ was measured using the IP-One kit (Cisbio) following the manufacturer’s instructions. Readings were measured with a SpectraMax i3 plate reader (Molecular Devices, Sunnyvale, CA). Each condition was run in triplicate, and experiments were repeated independently at least three times.

### cAMP Assay

CHO-K1 cells were seeded in 6 well plates and transfected with indicated plasmids and pGloSensor-22F. Cells were then lifted using TrypLE Express (Thermo Fisher Scientific) and resuspended in 1 ml DMEM/F12. 10 μl of cell suspension was transferred to wells of a white clear-bottom 384 well plate and left to adhere overnight. The next day, 10 μl of 1.2 mg/ml D-luciferin (GoldBio, CAS 115144-35) in CO_2_ Independent Medium was added to each well and allowed to incubate for a minimum of 2 h. A separate 384 well plate with peptide agonist, PK2, in CO_2_ Independent Medium at varying concentrations was prepared and placed in the FLIPR Tetra automated kinetic plate reader (Molecular Devices) together with the plate containing the cells. Luminescence was measured in every well at a sampling rate of 4 s. After 2 min of basal luminescence recording, agonist was injected and luminescence was measured for an additional 13 min. Each condition was run in triplicate, and experiments were repeated independently at least three times.

### Immunofluorescence Microscopy

CHO-K1 cells were seeded in six-well plates on glass coverslips and transfected with 2HA-PKR2 or mutant and either empty vector or MRAP2. The next day, coverslips were rinsed with PBS and fixed with 4% PFA in PBS for 10 min. Coverslips were rinsed three times, permeabilized cells were blocked with 5% non-fat dry milk in RIPA, non-permeabilized cells with 5% non-fat dry milk in PBS for 30 min at room temperature. Cells were then incubated with both mouse anti-HA at 1:5000 and polyclonal rabbit anti-MRAP2 at 1:5000 in 5% non-fat dry milk in PBS overnight at 4°C. Coverslips were rinsed three times with PBS and incubated with goat-anti rabbit Plus 555 (Invitrogen) and Alexa Fluor 488 goat-anti mouse (Invitrogen) in blocking buffer at 1:5000 dilution. Coverslips were rinsed three times and mounted on glass slides with SlowFade^TM^ Diamond Antifade Mountant with DAPI (ThermoFisher Scientific #S36963). Images were obtained with an inverted epifluorescence microscope (Olympus IX83). Experiments were acquired three times independently.

## Results

### PKR2 Is Glycosylated at N7 and N27

**FIGURE 1:**
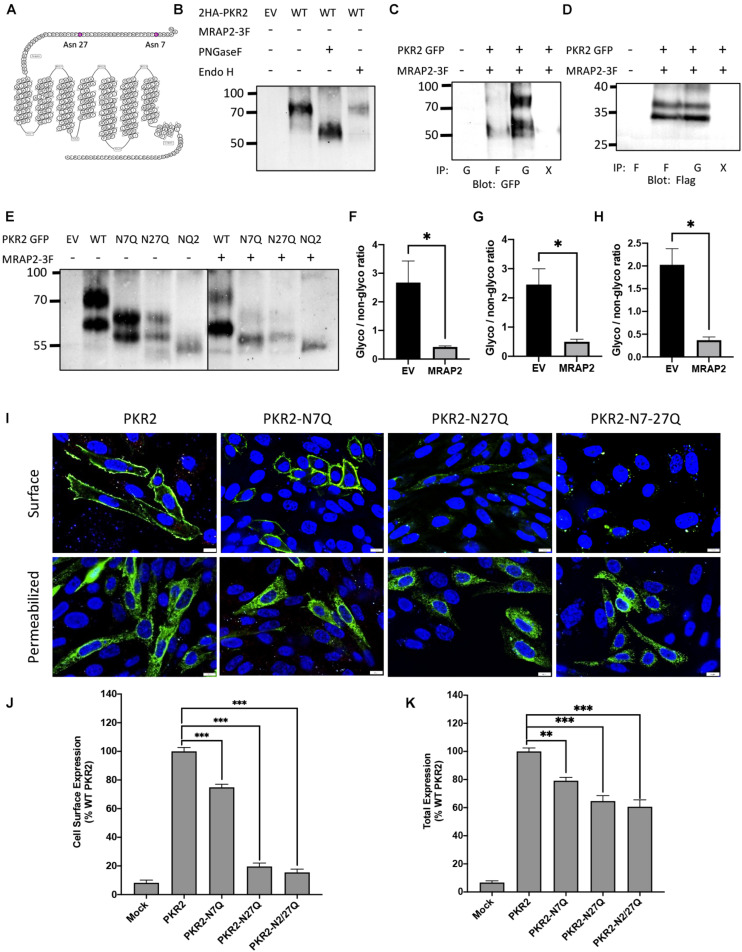
Role of N-linked glycosylation in PKR2 trafficking. **(A)** Schematic depiction of human PKR2 glycosylation sites. **(B)** Western blot detection of untreated PKR2-GFP or PKR2-GFP treated with PNGase F or EndoH. **(C,D)** Western blot detection of PKR2 **(C)** or MRAP2 **(D)** following co-immunoprecipitation of PKR2-GFP and MRAP2-3XFLAG from transfected CHO cells. Immunoprecipitation was performed using beads coated with GFP nanobodies “G” or anti-Flag antibody “F.” “X” indicates that no antibody was used for the IP (beads only). **(E)** Western blot detection of PKR2-GFP alone or with MRAP2 treated or not with PNGase F or Endo H. **(F–H)** Densitometry analysis measuring the ration of glycosylated to non-glycosylated PKR2 in the presence and absence of MRAP2 for WT **(F)**, N7Q mutant **(G)**, and N27Q mutant **(H)**. **(I)** Immunofluorescence detection of 2HA-PKR2, 2HA-PKR2-N7Q, 2HA-PKR2-N27Q, and 2HA-PKR2-N7/27 in non-permeabilized and permeabilized cells. **(J,K)** Cell ELISA detection of 2HA-tagged PKR2 and PKR2 mutants in non-permeabilized **(J)** and permeabilized **(K)** cells. Errors are mean ± SEM. ^∗^*p* < 0.05, ^∗∗^*p* < 0.01, ^∗∗∗^*p* < 0.001.

Based on the amino acid sequence, PKR2 has 2 N-linked glycosylation sites in the N-terminal region of the receptor at positions 7 and 27 (Figure 1A). To determine if those sites are in fact glycosylated, we transfected CHO cells with PKR2-GFP. Cells were lysed and PKR2 was immunoprecipitated using a GFP nanobody. Samples were then treated with either PNGase F or Endo H to remove mature or core glycosylation, respectively, before resolving proteins by SDS-PAGE and detecting PKR2 by western blot using anti-GFP antibody. Untreated PKR2 ran as a smear typical of glycosylated GPCRs at around 80–90 kD (Figure 1B). PNGase F treatment resulted in increased mobility of PKR2 consistent with deglycosylation (Figure 1B). The high molecular weight smear was, however, not affected by Endo H treatment, thus identifying this band as the mature glycosylated form of the receptor (Figure 1B). We had previously shown that PKR2 trafficking to the plasma membrane and signaling is inhibited by the accessory protein MRAP2. To test whether the interaction of MRAP2 with PKR2 results in a decrease in receptor glycosylation, cells were transfected with PKR2-GFP and MRAP2-3XFlag. Samples were incubated with either anti-GFP nanobody coated beads or with anti-Flag antibody to immunoprecipitate PKR2 or MRAP2, respectively. Samples were resolved by SDS-PAGE and proteins were detected by western blot using either anti-GFP or anti-Flag antibodies. In samples immunoprecipitated with the GFP nanobodies, the receptor separated in two bands (glycosylated and immature receptor), however, in samples immunoprecipitated with anti-flag antibody, the immature form of the receptor was enriched (Figure 1C), thus suggesting that interaction of MRAP2 preferentially interacts with the non-glycosylated form of PKR2 and possibly prevents its glycosylation. Co-immunoprecipitation of PKR2 and MRAP2 was also confirmed by detection of MRAP2 in samples immunoprecipitated with anti-GFP nanobodies (Figure 1D). To determine if both N7 and N27 can be glycosylated, we generated PRK2 constructs with substitution of N7 and/or N27 with a glutamine (N7Q, N27Q, and N7/27Q mutants). Mutants were transfected in CHO cells with or without MRAP2. Unmodified PKR2-GFP ran as two bands as described earlier (Figure 1E). The higher band representing the mature receptor and the lower band the core glycosylated receptor. Substitution of either N7 or N27 to Q resulted in increased mobility of both bands thus demonstrating that both sites can be glycosylated (Figure 1E). Substitution of both N7 and N27 to Q resulted in the complete loss of mature and core glycosylated receptor and only the immature non-glycosylated receptor was detected (Figure 1E). In the presence of MRAP2, the same profile is observed, however, in every case the relative amount of mature receptor compared to the immature form is greatly decreased (Figure 1E). Densitometric analysis shows a significant decreased ratio of glycosylated over non-glycosylated PKR2 in the presence of MRAP2 for WT PKR2 (Figure 1F), N7Q mutant (Figure 1G), and N27Q mutant (Figure 1H), thus further demonstrating that the presence of MRAP2 prevents PKR2 glycosylation at both N7 and N27 sites.

### Glycosylation at N27 Is Essential for PKR2 Trafficking

To assess the importance of glycosylation at N7 and N27 of PKR2 for receptor trafficking, PKR2, PKR2-N7Q, PKR2-N27Q, and PKR2-N7/27Q N-terminally fused to 2-HA tags were transfected in CHO cells. Localization of WT and mutant receptors was then determined by immunofluorescence microscopy in non-permeabilized and permeabilized cells using anti-HA antibody. Whereas PKR2 and PKR2-N7Q were readily detectable at the surface of non-permeabilized cells, PKR2-N27Q and PKR2-N7/27Q were not (Figure 1I). WT and all mutants were detected in permeabilized cells, thus confirming expression (Figure 1I). Results from this experiment suggest that glycosylation of N27 is essential for PKR2 trafficking to the plasma membrane. To quantitatively verify this finding, PKR2 and mutated PKR2 were transfected in CHO cells before measuring surface and total expression of receptor in non-permeabilized and permeabilized cells, respectively, by in-cell ELISA using anti-HA antibody. In agreement with the microscopy results, the N7Q mutant was readily detectable at the cell surface, albeit at a lower density compared to the non-mutated receptor (Figure 1J). In contrast, the surface density of N27Q and N7/27Q mutants were drastically reduced (∼90% decrease) compared to control receptor (Figure 1J). The total expression of mutated PKR2 measured in permeabilized cells was only reduced by 20–40% (Figure 1K), thus, the decrease in expression is not sufficient to explain the drastic loss of surface expression for PKR2-N27Q and N7/27Q. These results suggest that glycosylation at N27 is essential for receptor trafficking to the cell surface and may play a role in receptor stability.

### MRAP2 Prevents PKR2 From Trafficking to the Plasma Membrane Regardless of Glycosylation State

**FIGURE 2:**
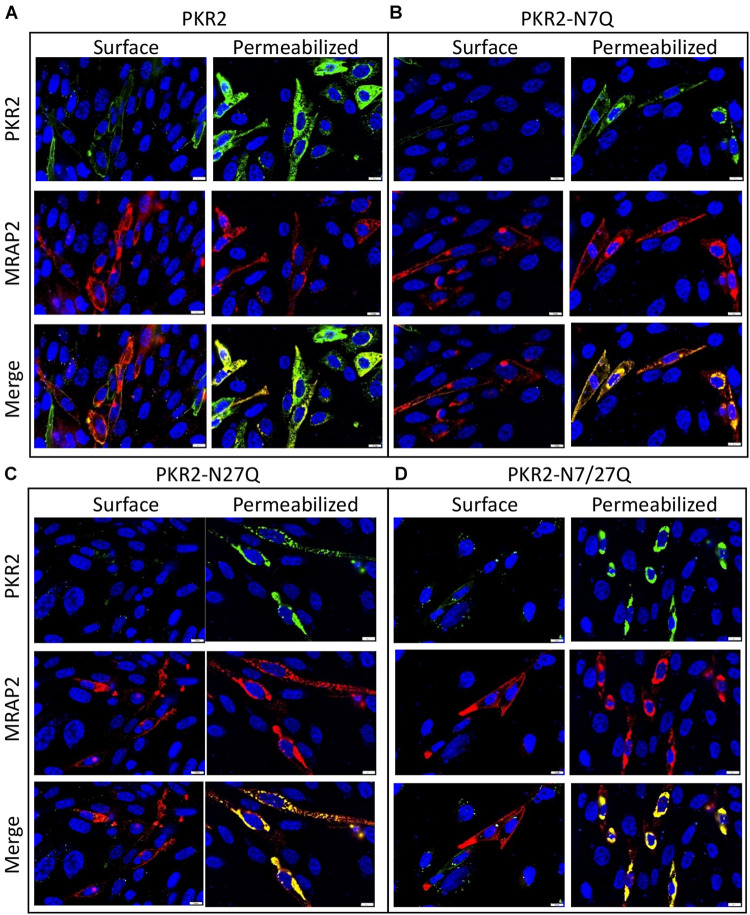
Localization of PKR2 and glycosylation mutants PKR2 expressed with MRAP2. **(A–D)** Immunofluorescence detection of MRAP2 and 2HA-PK2 **(A)**, 2HA-PKR2-N7Q **(B)**, 2HA-PKR2-N27Q **(C)**, and 2HA-PKR2-N7/27Q **(D)** in non-permeabilized or permeabilized cells. PKR2 is shown in green, MRAP2 is shown in red and yellow depicts co-localization. Nuclei are shown in blue (DAPI).

We had previously shown that surface density of PKR2 was reduced in the presence of MRAP2. Here we tested if receptor glycosylation alters the effect of MRAP2 on PKR2 localization. To this end, we performed immunofluorescence microscopy in CHO cells transfected with PKR2, PKR2-N7Q, PKR2-N27Q, or PKR2-N7/27Q in the presence of MRAP2. Whereas in the absence of MRAP2, PKR2, and PKR2-N7Q were detectable on the surface of non-permeabilized cells (Figure 1F), detection of those proteins was drastically reduced by MRAP2 expression (Figures 2A,B). For PKR2-N27Q and PKR2-N7/27Q, no receptor was detected at the plasma membrane regardless of MRAP2 expression (Figures 1F, 2C,D). Images of permeabilized cells show that MRAP2 causes a retention of PKR2 and PKR2-N7Q in intracellular compartments (Figures 2A,B). Additionally, MRAP2 staining displayed extensive co-localization with PKR2 and PKR2 mutants (Figures 2A–D).

**FIGURE 3:**
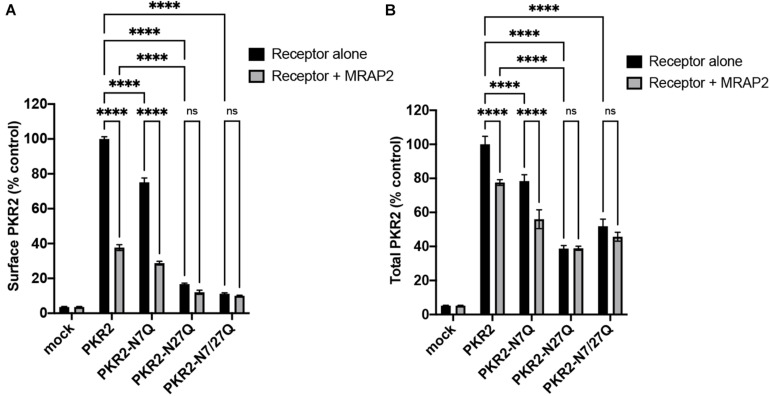
Surface and total quantitation of PKR2 and PKR2 mutants with and without MRAP2. **(A)** Cell ELISA detection of 2HA-PKR2 and 2HA-PKR2 mutants expressed alone or with MRAP2 in non-permeabilized cells. **(B)** Cell ELISA detection of 2HA-PKR2 and 2HA-PKR2 mutants expressed alone or with MRAP2 in permeabilized cells. Errors are mean ± SEM. ^*⁣*⁣**^p < 0.0001.

To quantitatively measure the effect of MRAP2 on the trafficking of PKR2 and glycosylation mutants of PKR2, 2HA-PKR2, 2HA-PKR2-N7Q, 2HA-PKR2-N27Q, and 2HA-PKR2-N7/27Q were transfected alone or with MRAP2 before measuring surface density and total expression by cell ELISA. Both WT and PKR2-N7Q were highly expressed at the cell surface and surface density was drastically reduced by MRAP2 for both (Figure 3A). Surface expression of PKR2-N27Q and PKR2-N7/27Q was very low regardless of MRAP2 expression (Figure 3A). Total expression of all receptor forms was readily detectable, however, expression of PKR2-N27Q and PKR2-N7/27Q were about half of the unmodified receptor (Figure 3B). MRAP2 expression caused a small decrease in total expression of PKR2 and PKR2-N7Q but did not alter the total expression of PKR2-N27Q and N-7/27Q (Figure 3B).

### Glycosylation Is Important for PKR2 Signaling

**FIGURE 4:**
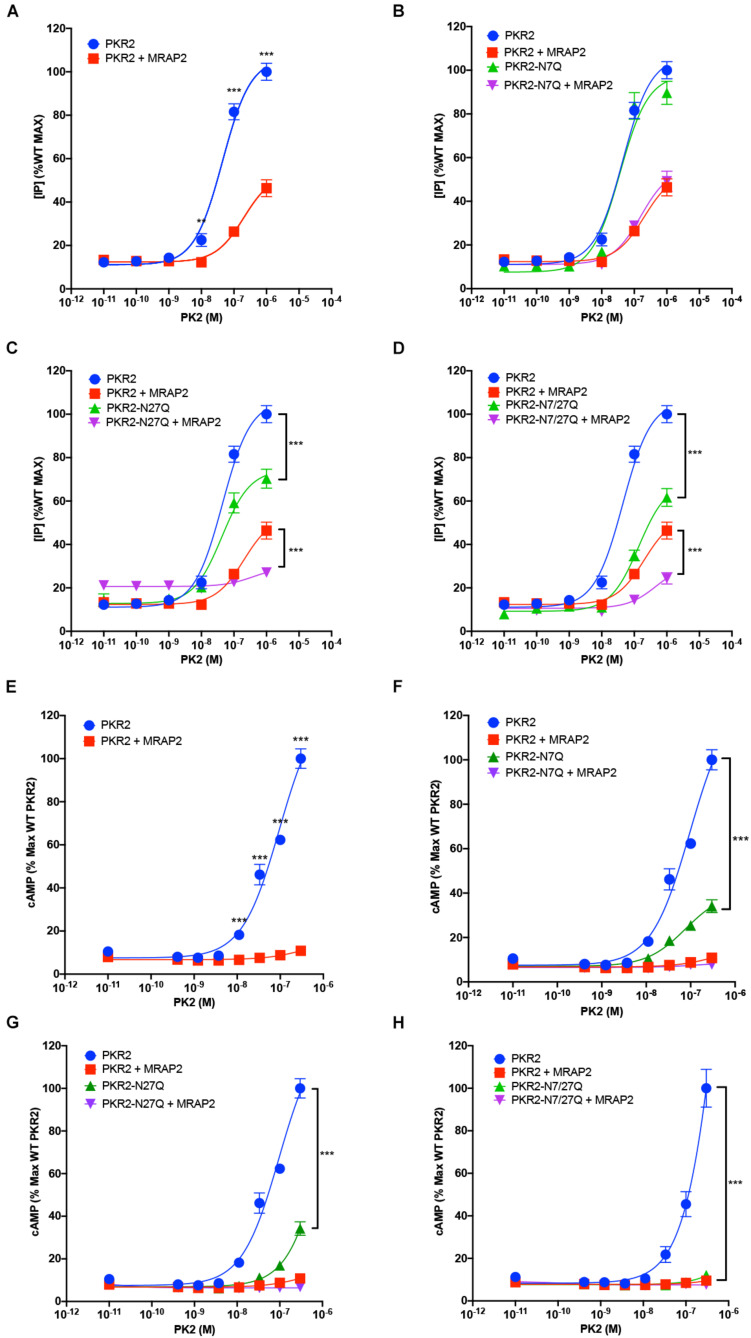
Role of N-glycosylation in PKR2 signaling. **(A–D)** Inositol phosphate detection in cells expressing PKR2 **(A)**, PKR2-N7Q **(B)**, PKR2-N27Q **(C)**, or PKR2-N7/27Q **(D)** with or without MRAP2 and stimulated with the indicated concentration of PK2. **(E–H)** cAMP measurement in cells expressing PKR2 **(E)**, PKR2-N7Q **(F)**, PKR2-N27Q **(G)**, or PKR2-N7/27Q **(H)** with or without MRAP2 and stimulated with the indicated concentration of PK2. Errors are mean ± SEM. **p < 0.01, ***p < 0.001.

Prokineticin receptors can signal through both Gα_q/__11_ and Gα_s_. To determine the importance of glycosylation for PKR2 signaling through Gα_q/__11_, we measured PK2-mediated IP3 production in CHO cells expressing either PKR2, PKR2-N7Q, PKR2-N27Q, or PKR2-N7/27Q in the presence or absence of MRAP2. PK2 caused a concentration dependent increase in IP3 production in cells expressing PKR2 (Figure 4A). As previously shown, MRAP2 drastically inhibited PKR2 signaling (Figure 4A). Remarkably, mutation of asparagine 7 did not alter PKR2 signaling through Gα_q/__11_ or the inhibition caused by MRAP2 (Figure 4B). In contrast, mutation of N27 (Figure 4C) or both N7 and N27 (Figure 4D) resulted in a significant decrease in PK2 efficacy and IP3 production, thus demonstrating that glycosylation at N27 is important for PKR2 function. The decrease in PK2 efficacy is likely due to the decrease in surface receptor density when N27 is not glycosylated. We then tested the effect of removing the N7 and/or N27 glycosylation sites on PKR2 signaling through Gα_s_ by measuring PK2-stimulated cAMP production. Here again, PK2 caused a concentration-dependent increase in cAMP production in cells expressing PKR2 and cAMP production was drastically inhibited by MRAP2 (Figure 4E). Interestingly, whereas the N7Q mutation had no adverse effect on PKR2-mediated IP3 production (Figure 4B), loss of glycosylation at N7 resulted in a significant decrease in PK2-stimulated cAMP production (Figure 4F), thus suggesting that glycosylation at N7 is not essential for signaling through Gα_q/__11_ but is important for Gα_s_ activation. MRAP2 further inhibited signaling of PKR2-N7Q. Gα_s_ signaling of both N27Q and N7/27Q mutants were greatly reduced (Figures 4G,H), in agreement with the large decrease in surface density of those mutants.

## Discussion

Prokineticin receptors play numerous important physiological roles like regulating feeding, pain or reproduction. Deletion of PKR2 in rodents results in multiple severe developmental phenotypes including underdeveloped olfactory bulbs, sterility, altered thermogenesis, and circadian rhythm reminiscent of Kallmann syndrome (Matsumoto et al., 2006). Like most GPCRs, PKR2 can be glycosylated at the N-terminus, however, the importance of this post-translational modification for PKR2 trafficking and signaling was not established. In many cases, N-linked glycosylation of GPCRs was shown to be important for receptor folding and trafficking to the plasma membrane (Karpa et al., 1999; Lanctot et al., 1999; Compton et al., 2002; Michineau et al., 2004; Wang et al., 2020; Patwardhan et al., 2021) whereas in others glycosylation appears non-essential (van Koppen and Nathanson, 1990; Fukushima et al., 1995; Karpa et al., 1999; Kozielewicz et al., 2017; Patwardhan et al., 2021). We identified two glycosylation sites on the N-terminal region of PKR2 and found that while glycosylation at position 27 is essential for PKR2 trafficking to the plasma membrane, glycosylation at position 7 is not. We had previously identified MRAP2 as an interacting protein of PKR2 and showed that MRAP2 sequesters PKR2 in intracellular compartments (Chaly et al., 2016; Rouault et al., 2017b). Here we show that in the presence of MRAP2, PKR2 glycosylation is largely prevented, which is consistent with the receptor being retained in the endoplasmic reticulum (ER). The lack of glycosylation of PKR2 in the presence of MRAP2 suggests that PKR2 is retained in the ER rather than trafficked to the membrane and rapidly internalized. MRAP2 prevented trafficking of both the unmodified and N7Q PKR2 mutants, thus suggesting that N7 is not important for MRAP2-mediated inhibition of PKR2. Mutation of N27 resulted in a decrease in both Gα_s_ and Gα_q/__11_ signaling, consistent with the decrease in surface receptor density. Surprisingly, however, mutation of N7 resulted in normal Gα_q/__11_ signaling but drastically reduced Gα_s_ coupling even though surface receptor density was only slightly reduced. This is particularly interesting because, whereas the importance of N-linked glycosylation for receptor trafficking has been reported for numerous GPCR, signaling bias as a result of a change in receptor glycosylation has only been identified for a few receptors (Marada et al., 2015; Soto et al., 2015; Patwardhan et al., 2021). Futures studies will need to be conducted to determine how glycosylation at position 7 modifies G-protein selectivity for PKR2 and the mechanism through which MRAP2 prevents PKR2 from exiting the ER.

## Data Availability Statement

The raw data supporting the conclusions of this article will be made available by the authors, without undue reservation.

## Author Contributions

JAV conducted experiments and wrote the manuscript. JAS supervised the projects and edited the manuscript. Both authors analyzed the data, contributed to the article, and approved the submitted version.

## Conflict of Interest

The authors declare that the research was conducted in the absence of any commercial or financial relationships that could be construed as a potential conflict of interest.

## Publisher’s Note

All claims expressed in this article are solely those of the authors and do not necessarily represent those of their affiliated organizations, or those of the publisher, the editors and the reviewers. Any product that may be evaluated in this article, or claim that may be made by its manufacturer, is not guaranteed or endorsed by the publisher.
